# Accuracy of Reaction Time Measurement on Automated Neuropsychological Assessment Metric UltraMobile

**DOI:** 10.1093/arclin/acae070

**Published:** 2024-09-13

**Authors:** Jacques Arrieux, Brian Ivins

**Affiliations:** Traumatic Brain Injury Center of Excellence, 5373 Gruber Road, Building C8837, Fort Liberty, NC 28310, USA; Womack Army Medical Center, Intrepid Spirit Center, 3908 Long Street Road, Fort Liberty, NC 28310, USA; General Dynamics Information Technology, 3150 Fairview Park Drive, Falls Church, VA 22042, USA; General Dynamics Information Technology, 3150 Fairview Park Drive, Falls Church, VA 22042, USA; Traumatic Brain Injury Center of Excellence, SSMC1, 1335 East West Highway, Silver Spring, MD 20910, USA

**Keywords:** Cognitive assessment, Reaction time, Mobile applications, Concussion screening, Psychometrics, Norms

## Abstract

**Objective:**

This observational study examined the accuracy of simple reaction time (RT) measurements on various touchscreen tablet devices using the Automated Neuropsychological Assessment Metric (ANAM) UltraMobile test battery. The study investigated the implications of interpreting ANAM UltraMobile with laptop-based normative data by analyzing the magnitude and variability of RT accuracy across devices.

**Method:**

RT accuracy on 10 different tablets was assessed using a photodetector and robotic arm to respond to stimuli at predetermined response times. The recorded RT was compared with the true RT obtained from the robotic arm to calculate the RT error.

**Results:**

ANAM UltraMobile recorded slower RTs than the laptop version. Additionally, RT error varied considerably among the 10 tablet models, suggesting psychometrically significant implications that could lead to interpretive errors when using laptop-based normative data.

**Conclusions:**

Relative to the RT error from the laptop-based version of ANAM, tablet data from ANAM UltraMobile are significantly slower and exhibit large variability between devices. These differences may have clinically significant implications for the comparability of the two versions. The findings suggest that further research with human participants is needed to assess the equivalence of ANAM UltraMobile with its predecessor.

## INTRODUCTION

Mobile cognitive tests have recently become widespread, and many commonly used computerized and paper-based assessment tools are now adapted for administration on mobile handheld touchscreen devices. Cognitive testing with mobile devices offers potential advantages over legacy formats, such as sub-second reaction time (RT) measurement, reduced equipment costs, increased portability, and large group administration (Bauer & Bilder, 2023). Changing testing modalities also introduces measurement challenges, notably in comparing performance between legacy and mobile formats (Bauer et al., 2012; Bauer & Bilder, 2023; Cernich et al., 2007; Kane & Parsons, 2019; Parsons & Duffield, 2019; Schatz et al., 2015). Novel computerized adaptations are often considered different tests from their predecessors and require updated normative data for interpretation (American Psychological Association, 2020; Bauer et al., 2012).

Factors confounding the interpretation of mobile cognitive tests include variability in device specifications (e.g., hardware, software, and Operating System [OS]) and differences in response modality (e.g., pencil, mouse, touchscreen; Germine et al., 2019). Because many mobile cognitive tests measure responses at the sub-second level, the precision of RT measurements is among the most salient issues affecting the clinical utility of cognitive testing on mobile devices. For example, Schatz et al. (2015) found an approximate 20–100 millisecond (ms) range of RT errors on various tablet devices (i.e., iPad, Kindle Fire, Google Nexus, and Samsung Galaxy). The authors noted that the implications of such findings could be a significant barrier to the interpretation of mobile cognitive tests, as RT measurement error can account for up to 40% of the range of simple RT responses from study participants (Schatz et al., 2015). Importantly, these findings indicate that depending on the mobile device used to administer the test and the magnitude of its error, a participant’s performance could be misinterpreted as abnormally slow and potentially misinform clinical decision-making. The susceptibility of these measures to be confounded by variable RT measurement accuracy has led to growing concerns about how clinicians should use and interpret these novel measures, particularly regarding the continued use of legacy normative data versus the development of new reference groups.

The concern that changes in testing format can impact clinical interpretation has been addressed in several studies that have evaluated the comparability of performance across testing modalities (Backx et al., 2020; Brearly et al., 2019; Buckley et al., 2017; Corcoran, 2022; Cyr et al., 2021; Elbin et al., 2020; Kuzmuk et al., 2022; Mielke et al., 2015; Ott et al., 2022; Stricker et al., 2019; Vermeent et al., 2022; Young, 2020). Many of these studies showed a clinically meaningful effect of the administration method on performance measurements, and these differences have been addressed by creating new normative data or by applying adjustments to existing normative data. However, despite an understanding that adapting tests to mobile devices introduces novel sources of error, developers have rapidly transitioned tests to tablet administration and often use normative data derived from legacy formats (Immediate Post Concussion Assessment Test [ImPACT], Pearson Q-Interactive (Wechsler Adult Intelligence Scale, fifth edition [WAIS-IV], Wechsler Memory Scale, fourth edition [WMS-IV], California Verbal Learning Test, third edition [CVLT-III], Delis Kaplan Executive Functioning System [DKEFS]), and National Institutes of Health Toolbox Cognitive Battery) (Daniel, 2012; Gershon et al., 2017; Wallace et al., 2020; Young, 2020).

Brief computerized cognitive tests are widely used to screen for concussions in athletes and military service members (Alsalaheen et al., 2016; Jones et al., 2021). The U.S. military has primarily used the Automated Neuropsychological Assessment Metric, fourth edition, TBI Military Battery (ANAM4-TBI-MIL) (Belanger et al., 2022; Department of Defense, 2015; Jones et al., 2021; Meyers & Vincent, 2020; Porter & Johnson, 2020; Reeves et al., 2007; United States Special Operations Command, 2019). The DoD ANAM4-TBI-MIL data repository includes pre-deployment baseline data from over one million active-duty service members, which serve as references for post-injury assessment comparisons and as normative data when baselines are not available for a patient (Haran et al., 2016; Meyers & Vincent, 2020; Porter & Johnson, 2020). Because computerized cognitive tests such as ANAM are susceptible to machine-based sources of measurement error (e.g., hardware, OS, or other technical specifications), test administration is standardized to specific computer platforms to ensure reliable comparisons with normative or baseline data (Arrieux et al., 2020; Cernich et al., 2007; Rice et al., 2011; Schlegel & Gilliland, 2007; Vista Life Sciences, 2015). The original laptop model on which the ANAM normative data were developed (Dell D630, released in 2007) is approximately 30 ms slower than the actual RTs. The D630 laptop is now obsolete and no longer available for purchase; therefore, data from newer laptop models are adjusted to remain equivalent to data from the normative sample. For example, the model currently being used (Dell E6440, released in 2013) records RT at approximately 70 ms slower than true RTs, requiring a − 40 ms adjustment to be compatible with normative data generated on the D630 (Vista Life Sciences, 2015).

In recent years, in line with broader trends in cognitive assessment, ANAM4-TBI-MIL was adapted for administration on Samsung tablets, called ANAM UltraMobile (Vincent et al., 2019). This tablet-based version is designed to be nearly identical to its computer-based predecessors, including the same subtests, stimuli, interstimulus intervals, and scores (Vincent et al., 2019; Vista Life Sciences, 2022a). The new iteration of the test changes the response modality from an external computer mouse with the index and middle fingers of the dominant hand to 8″ or 10″ handheld touchscreen tablets on which examinees respond with the thumbs of both hands.

Given that a large military normative dataset for the laptop version of ANAM is available, using those existing laptop-based normative data and pre-injury baseline assessments could expedite the clinical use of ANAM UltraMobile in the military rather than delaying its use until new tablet-based normative data are developed and baselines are obtained. To accomplish this, ANAM UltraMobile data are *adjusted* to make them equivalent to existing laptop-based normative and baseline data for clinical interpretation. Specifically, ANAM UltraMobile software automatically applies subtest-specific adjustments to trial-by-trial level responses to equate performance with the laptop-based performance data. However, the comparability of performance across the two platforms or the validity of the equating adjustments has yet to be published in test manuals or peer-reviewed literature.

Utilizing normative and baseline data from legacy versions of cognitive tests to interpret versions administered on handheld tablets requires cross-platform studies to determine whether there is sufficient psychometric comparability between platforms to use the legacy data and to validate any adjustments that may be applied to achieve comparability. This study is the first of a series of studies intended to assess the comparability of ANAM performance across administration platforms and to appraise the validity of the current score adjustment proposed by the test developer. This study aimed to examine the accuracy of ANAM UltraMobile RT measurement across various tablet models and assess the potential clinical implications of any observed differences. Specifically, this study evaluated the accuracy of RT measurements on ANAM UltraMobile Simple Reaction Time (SRT) and Simple Reaction Time Repeated (SR2) subtests on ten tablet specifications using a photodetector and Robotic Key Actuator (RKA) to respond to stimuli at predetermined response speed intervals. The recorded RT from ANAM UltraMobile was compared with the true RT obtained from the robotic arm to calculate RT latency. The magnitude and variability of RT accuracy across devices were used to explore the implications of interpreting ANAM UltraMobile with laptop normative data.

## METHODS

### ANAM UltraMobile

Adapted from its laptop-based predecessor, ANAM UltraMobile is a brief computerized cognitive test battery designed to assess constructs impacted by concussive injury using a Samsung handheld touchscreen tablet (Vincent et al., 2019; Vista Life Sciences, 2022a). Examinees hold the tablet with both hands and use their thumbs to touch the designated response icons on the screen. ANAM UltraMobile uses the same battery of 10 subtests as the military laptop version, which involves simple and choice RT tasks designed to assess factors such as processing speed, attention, working memory, learning, and recall. Additionally, ANAM UltraMobile produces scores identical to the laptop version for each subtest: mean RT per correct response, percent correct, and throughput (number of correct responses per minute of available test time). The program uses normative data to automatically convert raw scores into standardized scores, generate percentile rankings, and reliable change parameters (Vista Life Sciences, 2015).

### Touchscreen tablets

This study used ten touchscreen tablet models running Android OS (Table 1). Four tablets were selected based on developer recommendations (Vista Life Sciences, 2022a). Because some devices certified to run the test were obsolete when data collection began, an additional six comparable tablets were included to further examine RT accuracy across multiple generations of tablet models that could be used for ANAM UltraMobile administration.

**Table 1:** Tablet models used in the study

Device Name	Model number	Year released	OS	Display size
Samsung Galaxy Tab A 8.0	SM-T350^a^	2015	Android 7	8.0”
Samsung Galaxy Tab A 8.0	SM-T380^a^	2017	Android 9	8.0”
Samsung Galaxy Tab A 10.1	SM-T510	2019	Android 9	10.1”
Samsung Galaxy Tab A 8.0	SM-T290	2019	Android 11	8.0”
Lenovo Tab M8 HD	TB-8505F	2019	Android 9	8.0”
Samsung Galaxy Tab A7 Lite	SM-T220^a^	2021	Android 11	8.7”
Samsung Galaxy Tab Active3	SM-T570	2021	Android 12	8.0”
Samsung Galaxy Tab A8	SM-X200^a^	2022	Android 11	10.5”
Samsung Galaxy Tab A8	SM-X200	2022	Android 12	10.5”
Samsung Galaxy Tab S8	SM-X700	2022	Android 11	11”

^a^Tablet model certified by test developer (Vista Life Sciences, 2022a).

### Apparatus

The Black Box Tool Kit (BBTK) v2 produced ANAM UltraMobile data. BBTK is a computer-operated system designed to support the precise calibration of cognitive testing paradigms by using an optic sensor to detect stimuli on the tablet screen and a Robotic Key Actuator (RKA) to respond with automatic screen touches (Plant et al., 2004; Plant & Turner, 2009; Plant, 2015). This process supported the precise delivery of responses at predetermined RT intervals. The BBTK was preprogrammed to respond to SRT and SR2 stimuli at consistent timing intervals.

Based on prior research investigating RT measurement error on tablet devices (Schatz et al., 2015), which found statistically significant differences between RT accuracy at different RT intervals, the BBTK was preprogrammed to respond to ANAM stimuli at four RT intervals (i.e., 150, 300, 500, and 1000 ms). Using the four preset RT intervals generated a range of RTs that simulated the mean response times from the laptop military normative data and supported an examination of the accuracy of RT measurements at different response speeds. Additionally, based on prior research involving RT measurement accuracy on the laptop version of ANAM using the BBTK (Arrieux et al., 2020), four administrations of the SRT and SR2 subtests were included for analyses at each RT interval to account for potential variability in BBTK responses due to software glitches that resulted in the RKA not producing responses for all stimuli in the subtests.

While the BBTK supports consistent RTs at the microsecond level, two key issues were considered when choosing the number of trials: 1) the ANAM UltraMobile software was expected to crash midway through data collection occasionally, and 2) after repeated screen presses, the electrically conductive foam pad on the RKA that pressed the screen was expected to wear down which would produce a small number of trials with responses not registered by the ANAM UltraMobile application. These issues were mitigated by restarting the ANAM UltraMobile application following each subtest and replacing the foam pad after testing each device (i.e., replacing the foam pad after approximately 1280 responses). Each administration of the SRT and SR2 tests consisted of 40 trials, generating 40 responses per administration. This resulted in 1280 responses per tablet (40 trials × 4 administrations × 4 response intervals × 2 tests per tablet) and a total sample size of 12,800 responses across all tablet models studied. Due to anticipated software and hardware glitches described above that produced subtests with missing responses, 17 SRT (10.63%) and 14 SR2 (8.75%) subtests were repeated to produce the total sample size of 12,800 trials.

The SRT and SR2 subtests were selected because they use the same stimulus, which is a single color that consistently appears in the same location on the tablet screen, ensuring that the BBTK detects and responds to stimuli on every trial. Stimuli for other ANAM tests are more visually complicated, consisting of multiple shapes, colors, and locations. These were difficult to consistently detect with the BBTK because it used a fixed location photodetector.

### Analyses

RT error was calculated with three steps: 1) remove the ANAM UltraMobile software’s automatic score adjustments from each trial (i.e., −20 ms for SRT and −40 ms for SR2) to obtain the recorded RT. 2) Subtract the pre-programmed BBTK RT response times (i.e., 150, 300, 500, or 1000 ms) from the ANAM UltraMobile recorded RTs. 3) Calculate ANAM UltraMobile RT error relative to the laptop-based version of ANAM by subtracting 35 ms from each trial. Expressing tablet RT errors relative to the laptop ensured that all RT errors were scaled to a single reference, consistent with laptop-based military normative data, the intended comparator for assessing ANAM UltraMobile performance.

Next, the distributions of RT error of the tablets were examined using descriptive statistics (i.e., mean, standard deviation, and interquartile range [IQR]). Mean RT error from the tablets was then compared with the standard deviation (SD) of the mean RT for correct responses (RTC) from the laptop-based military normative data and expressed as a proportion of the normative RTC SD. This is equivalent to a z-score, a commonly used numeric scale expressing raw scores in standard deviation units. Z-scores are widely used in statistics and clinical practice to standardize continuous scores from different numeric and measurement scales into one that eases interpretation and facilitates comparison of diverse measures (Lezak et al., 2012). This approach provided a clinically meaningful effect size measure of tablet RT error because we used SDs from laptop-based military normative data intended to be used to interpret ANAM UltraMobile performance.

Inferential statistics were not used in our analysis for three reasons. First, the responses from the BBTK were equal at the ms level, indicating no variance in BBTK response times across individual ANAM UltraMobile trials. Second, the variability in RTs observed across individual ANAM UltraMobile trials was due to residual variability in the processing speeds of the tablets we tested. Using standard errors based on this variability to calculate test statistics, such as for a t-test or an omnibus F-test in an analysis of variance, would be misleading because this variability was many times smaller than the human variability in ANAM’s military normative data. These smaller standard errors artificially increased the likelihood of statistical significance, which would have been incompatible with test statistics calculated with the larger standard errors based on variability in ANAM RTs obtained from human test participants. Third, statistically significant differences in RT error between tablets would not necessarily have equated to clinically significant differences. Therefore, a statistically significant finding would merely indicate a low probability of RT error across various tablet models being exactly equal in the population of all tablets based on the data in our study sample.

## RESULTS

Table 2 shows the descriptive statistics for RT error between ANAM UltraMobile and the laptop version for the SRT and SR2 tests from various tablets. The mean RT errors on the SRT and SR2 tests vary considerably between tablet models and Android OSs. For example, the smallest RT errors for the SRT and SR2 subtests were 19.35 and 19.25 ms, respectively, on the TB-8505-F. The largest RT measurement error was found on the SM-T290 (SRT = 89.61 ms, SR2 = 88.42 ms). The mean RT errors on the TB-8505-F were equal to 0.42 and 0.44 laptop normative RTC SDs, while the SM-T290 errors were equal to 1.95 and 2.00 laptop normative RT SDs for the SRT and SR2 tests, respectively. The results also showed a notable increase in RT measurement errors with the same SM-X200 tablet after updating Android OS 11 to 12. Using Android OS 11, the errors for SRT were 30.31 and 30.14 for SR2. With the SM-X200 using Android OS 12, errors increased to 80.81 ms for SRT and 80.85 ms for the SR2 subtest. These RT errors corresponded to 0.66–0.68 laptop normative RTC SDs for Android 11 and 1.76–1.83 laptop normative RTC SDs for Android 12.

**Table 2:** Distribution characteristics of tablet RT error: RT error (ms) of ANAM UltraMobile relative the laptop version by tablet model

Tablet model	Subtest	RT Error m (sd)	Interquartile Range	Range 10^th^ to 90^th^ %ile	Full Range	Mean tablet RT error^a^
SM-T350^b^	SRT	55.80 (9.85)	13.75	26.00	62.00	1.21
	SR2	56.39 (9.99)	13.00	26.00	54.00	1.28
SM-T380^b^	SRT	40.72 (7.27)	10.00	18.00	51.00	0.88
	SR2	41.26 (7.22)	10.00	17.90	48.00	0.94
SM-T510^b^	SRT	28.38 (6.11)	8.00	15.90	43.00	0.62
	SR2	28.42 (6.15)	9.00	16.00	45.00	0.64
SM-T290	SRT	89.61 (9.16)	13.00	23.00	86.00	1.95
	SR2	88.42 (9.19)	13.00	24.00	46.00	2.00
TB-8505-F	SRT	19.35 (7.15)	9.00	18.90	59.00	0.42
	SR2	19.25 (7.10)	9.00	20.00	37.00	0.44
SM-T570	SRT	38.98 (8.48)	10.00	21.00	54.00	0.85
	SR2	39.03 (7.87)	10.00	20.00	54.00	0.88
SM-T220^b^	SRT	29.66 (5.99)	7.00	15.00	38.00	0.64
	SR2	29.82 (6.11)	7.00	16.00	41.00	0.68
SM-X200-OS11^b^	SRT	30.31 (4.78)	9.00	13.00	23.00	0.66
	SR2	30.14 (4.88)	8.00	14.00	25.00	0.68
SM-X200-OS12^b^	SRT	80.81 (5.00)	9.00	13.00	21.00	1.76
	SR2	80.85 (5.08)	8.00	14.00	24.00	1.83
SM-X700	SRT	51.83 (9.50)	11.00	23.00	71.00	1.13
	SR2	51.25 (9.50)	11.00	23.00	69.00	1.16

^a^As a proportion of 2019 military normative RTC SDs.

^b^Tablet model certified by test developer (Vista Life Sciences, 2022a).

The dispersion in the distributions of RT error was relatively small in all tablet models and consistent between models. For example, the smallest dispersion of distributions of RT errors were 4.78 and 4.88 ms on the SM-X200 running the Android 11 OS. In contrast, the largest was 9.85 and 9.99 ms on the SM-T350, a maximum difference of 5.11 ms between all tablet models. Dispersion between tablets was consistent, as indicated by similar IQRs of RT errors across devices. The SM-T220 model exhibited the smallest IQRs, with both SRT and SR2 subtests measuring 7.00 ms. In contrast, the IQRs for the SM-T350 model were 13.75 ms for SRT and 13.00 ms for SR2, reflecting a maximum IQR difference of 6.75 ms between devices. Furthermore, the mean RT errors and their dispersions were nearly identical within each tablet device for the SRT and SR2 subtests.

Table 3 shows the mean RT errors from the SRT and SR2 subtests between ANAM UltraMobile and the laptop version on different tablets by the four preset timing intervals. Except for the TB-8505-F model, the inter-interval differences in RT errors at different BBTK response intervals ranged from less than 1 ms to a maximum of 5.76 ms for both subtests. Notably, the largest inter-interval differences of RT errors, 11.26 ms for SRT and 11.51 ms, occurred between the 300 and 500 ms response intervals on the TB-8505-F tablet. However, the inter-interval differences in RT error between the 150, 500, and 1000 ms response intervals on the TB-8505-F only ranged from 0.11 to 2.74 ms for the SRT test and from 0.44 to 3.66 ms for the SR2 test, which is more consistent with the range of inter-interval error differences observed on other tablet models.

**Table 3:** RT error (ms) between tablets and laptop by ANAM test and response interval

Tablet model	Test	BBTK response interval				Mean RT difference from norms^a^ (z-score)
		150 ms m (sd)	300 ms m (sd)	500 ms m (sd)	1000 ms m (sd)	
SM-T350^b^	SRT	55.28 (10.05)	53.40 (9.64)	57.41 (10.02)	57.11 (9.22)	1.16–1.25
	SR2	58.97 (11.22)	53.21 (8.59)	56.83 (9.64)	56.56 (9.54)	1.21–1.34
SM-T380^b^	SRT	41.18 (8.40)	41.40 (6.93)	40.11 (6.65)	40.20 (6.96)	0.87–0.90
	SR2	40.47 (8.22)	41.96 (6.89)	41.67 (6.70)	40.94 (6.92)	0.92–0.95
SM-T510^b^	SRT	26.00 (5.99)	29.54 (6.82)	29.04 (5.71)	28.94 (5.20)	0.56–0.64
	SR2	25.29 (5.72)	30.23 (6.22)	28.38 (5.49)	29.77 (5.95)	0.57–0.69
SM-T290	SRT	89.90 (8.66)	91.01 (10.19)	88.01 (8.99)	89.53 (8.53)	1.91–1.98
	SR2	88.60 (10.39)	89.78 (8.28)	87.36 (8.82)	87.93 (9.03)	1.98–2.03
TB-8505-F	SRT	20.14 (6.15)	11.62 (4.64)	22.88 (5.80)	22.77 (5.25)	0.25–0.50
	SR2	19.49 (6.79)	11.64 (4.62)	23.15 (5.18)	22.71 (4.75)	0.26–0.52
SM-T570	SRT	39.77 (7.79)	38.25 (7.95)	36.96 (8.12)	40.93 (9.49)	0.80–0.89
	SR2	38.40 (6.06)	38.80 (7.97)	38.19 (9.24)	40.73 (7.72)	0.87–0.92
SM-T220^b^	SRT	31.34 (5.64)	29.93 (5.48)	28.93 (6.33)	28.43 (6.11)	0.62–0.68
	SR2	31.28 (5.72)	30.36 (5.97)	29.19 (6.13)	28.46 (6.28)	0.65–0.71
SM-X200-OS11^b^	SRT	30.17 (4.76)	30.44 (4.78)	30.24 (4.71)	30.38 (4.89)	0.66–0.66
	SR2	30.13 (4.80)	30.19 (4.96)	30.16 (4.81)	30.07 (4.98)	0.68–0.68
SM-X200-OS12^b^	SRT	81.56 (4.81)	80.43 (4.52)	80.54 (5.54)	80.71 (5.03)	1.75–1.77
	SR2	81.74 (4.76)	80.57 (4.57)	80.90 (5.48)	80.19 (5.37)	1.82–1.85
SM-X700	SRT	53.24 (9.54)	50.66 (10.3)	52.65 (9.72)	50.77 (8.11)	1.10–1.16
	SR2	49.53 (8.91)	52.08 (9.59)	51.82 (9.57)	51.59 (9.77)	1.12–1.18

^a^As a proportion of 2019 military normative RTC SDs

^b^Tablet model certified by test developer (Vista Life Sciences, 2022a).

Table 3 also shows that, despite their variability, the range of RT errors from different BBTK response intervals is a substantial proportion of the laptop normative RTC SDs for the SRT and SR2 tests. For example, the mean RT errors across the four intervals on the TB-8505-F, the tablet with the smallest RT errors relative to the laptop, were equal to 0.25–0.50 and 0.26–0.52 of the laptop normative RTC SDs for the SRT and SR2 tests. The mean RT errors across the four BBTK response intervals from the tablet with the largest RT errors relative to the laptop were equal to 1.91–1.98 and 1.98–2.03 of the laptop normative RTC SDs for the SRT and SR2 tests, respectively.

## DISCUSSION

This study found that ANAM UltraMobile recorded slower RTs than laptops. The results also demonstrated that RT errors were highly variable across tablet models and Android OS versions. Furthermore, RT errors were sizable in relation to laptop normative RTC SDs. The results suggest that tablet-based ANAM UltraMobile and laptop-based ANAM4-TBI-MIL scores based on RT may not be directly comparable. Variability in RT measurement accuracy across tablet models and Android OS versions indicates that a single universal RT adjustment may not be appropriate. Taken together, these results suggest that RT equating adjustments that do not fully account for tablet- and Android OS-specific RT errors could lead to notable interpretive errors on some subtests (e.g., SRT and SR2) and to a lesser extent on other subtests (e.g., Code Substitution or Mathematical Processing) when using laptop-based normative data as a reference. However, without human data, the full psychometric equivalence of the ANAM UltraMobile and laptop ANAM4-TBI-MIL remains unknown.

### Psychometric implications of device differences

This study revealed highly variable RT errors between tablet models, suggesting potential psychometric and clinical significance. Commonly used z-score cutoffs for interpreting performance can help elucidate interpretive implications (z ≤ −2.0 very superior, > − 2.0 to ≤ −1.30 superior, > −1.30 to <−0.60 high average, ±0.60 average, >0.60 to <1.30 low average, ≥1.30 to <2.0 borderline/unusually low, and ≥ 2.0 extremely low (Lezak et al., 2012). For example, an individual with laptop SRT and SR2 performance in the *average* range (z = 0.00) could be misinterpreted as *low average* on the SM-X200 with OS 11 (SRT z = 0.66 and SR2 z = 0.68) and *borderline/unusually low* with the SM-X200 with OS 12 (SRT z = 1.76 and SR2 z = 1.83). Using laptop-based normative data could result in interpretive errors if tablet-specific RT errors are not considered. For example, approximately 50% of a population falls within 0.66 SDs (−0.66 ≥ z ≤ 0.66) above or below the mean in a standard normal distribution. This means that approximately 25% of the population is within 0.66 SDs above the mean (i.e., between z > 0 and z ≤ 0.66). An RT error equal to 0.66 of the normative sample SD that is not accounted for will make 25% of the population of test takers who would otherwise be in the lower half of the average performance range on the laptop appear to be in the low average range on the SRT test if taken on the SM-X200 running Android v11. Individuals whose performance would otherwise be below the average range on the laptop would be moved even lower into the low average range or the borderline/unusually low range.

### Implications of using RT-based adjustments

A notable finding of this study was that the equating adjustments proposed by the test developer do not appear to account for RT error differences between tablet devices, as variable residual RT error remained after deducting the subtest-specific adjustments. This was a critical and unexpected finding because the equating adjustments were unknown before beginning our study. Considerable differences were found between equating adjustments and tablet RT errors in magnitude and direction. In addition, the differentials between the equating adjustments and the observed RT errors suggest that there may be some critical psychometric differences between ANAM UltraMobile and the laptop version resulting from both the change in the user interface from a mouse to a handheld touch screen and the hardware/software-based RT errors. These differences raise questions about the validity of the proposed RT equating adjustments that require further research before the equating adjustments are used. They also raise questions about whether equating scores from the two ANAM versions is feasible.

Using the SRT and SR2 subtest errors obtained across devices, an additional supplemental analysis was completed for all subtests in the battery to explore the remaining RT error after accounting for tablet-specific error (Supplemental Table 1 and Supplemental Fig. 1). The residual differential values were then expressed as a proportion of the laptop normative RTC SD for each ANAM subtest. Supplemental Table 1 shows the current ANAM UltraMobile RT equating adjustments and possible psychometric implications. The equating adjustments ranged from −53 ms for the GNG subtest to +125 ms for the M2S subtest. The adjustments for three subtests (SRT, SR2, and GNG) were negative, whereas the remaining subtests were positive. The negative equating adjustments imply that human test takers performed slower, and the positive equating adjustments imply that human test takers performed faster on tablet-based ANAM UltraMobile. Supplemental Table 1 and Supplemental Fig. 1 also show that the current equating adjustments do not fully account for the RT error between various tablet models and laptops. For example, the 20 ms RT equating adjustment for the SRT test does not account for 10.3 ms of RT error on the SM-X200 tablet running the Android v11 OS, which equals 0.22 SDs for RTC in the laptop-based military normative data. On the SM-X200 tablet running Android v12, the 20 ms RT equating adjustment does not account for 60.8 ms of RT error, which equals 1.32 SDs for RTC in the laptop-based military normative data. The last column of Supplemental Table 1 shows the magnitude of the unaccounted-for error across all ANAM UltraMobile tests relative to the SD for RTC in the laptop-based military normative data. For some tests, such as mathematical processing, the error unaccounted for by the current equating adjustment only ranges from −0.11 to −0.02 laptop normative RTC SDs. These are trivial to small effects and would only result in minimal interpretive error. However, for the SRT, SR2, Procedural RT, and Go No-Go subtests, the RT error unaccounted for by the current equating adjustment on some tablet models could be equal to one or more laptop normative RTC SDs, which are large and would result in a sizable number of interpretive errors.

RT is not the only ANAM score that could be affected by platform differences. ANAM produces other scores, such as the number of lapses (timeout), number of bad responses (anticipatory responses), and percent correct, which are used to calculate the throughput score most often used for clinical interpretive purposes. These scores could be affected by changing the user interface from a computer mouse to a touch screen. However, no available information demonstrates whether these scores are similar to those of ANAM UltraMobile and its laptop-based predecessor, on which the existing normative data were obtained. Furthermore, there could be differences in practice and fatigue effects between the two ANAM versions resulting from the change in the user interface, but no peer-reviewed literature has demonstrated whether scores are similar or different. The variable equating adjustments, especially the different directions of the adjustments shown in Supplemental Table 1, suggest that performance on ANAM UltraMobile appeared faster than on the laptop for seven of the ten tests despite our finding that all of the tablet models we tested had slower processing speeds than the laptop. These findings suggest that psychometric differences other than machine-based RT error might exist between ANAM UltraMobile and its laptop predecessor. Further research comparing performance differences across all subtests is required to determine whether the performance of ANAM UltraMobile can be interpreted using normative data obtained on the laptop.

Developing RT equating adjustments for specific tablet models and OS versions might initially appear straightforward, but this study elucidates the complexity of some of the problems involved. This study showed that mean RT errors for the SRT and SR2 tests were nearly identical within the same tablet with nearly identical standard deviations. This suggests RT errors are constant across ANAM tests within the same tablet model and OS. However, this study did not find any evidence of device-specific equating adjustment.

The results of this study suggest that the psychometric implications of adjustment require further examination. First, the equating adjustments for seven ANAM UltraMobile tests were positive, suggesting that examinees will generate faster RTs on ANAM UltraMobile than on the laptop. This was surprising, considering all the tablets examined were slower than the laptop. Second, if ANAM UltraMobile recorded faster human RTs on some tests than the laptop version, thereby requiring an adjustment that is added to ANAM UltraMobile’s recorded RT to equate it to a laptop RT, then the equating adjustment may be a net numeric adjustment that does not fully account for the RT error (see Supplemental Table 1 and Supplemental Fig. 1 for further illustration of this example). If there were no performance differences between ANAM UltraMobile and the laptop version resulting from the change in the user interface from a mouse to a handheld touchscreen device, the equating adjustments for every ANAM UltraMobile test should have been equal to device-specific RT error, but they were not. Third, despite the two tests being identical, the RT equating adjustment for the SR2 test (−40 ms) was twice as large as the equating adjustment for the SRT subtest (−20 ms). This implies that examinees are expected to perform slower on SR2 than on the SRT test, suggesting a possible 20 ms fatigue effect because SR2 is the seventh of ten subtests administered on both battery versions. These differences between SRT and SR2 on ANAM UltraMobile are inconsistent with laptop-derived military normative data, where the raw mean RT correct for the SRT and SR2 are 261.964 and 261.661 ms, respectively. A difference of −0.303 ms is psychometrically negligible; however, the +20 ms difference on ANAM UltraMobile is equal to 0.43 and 0.45 RTC SDs from the laptop-derived normative data for those two tests. Finally, if the change in the user interface results in different practice or fatigue effects on ANAM UltraMobile than on the laptop version, the sensitivity of ANAM UltraMobile to detect cognitive sequelae could differ from that of the laptop version.

### Implications for practical use

Variable RT errors due to hardware and software differences between tablet models could compromise the reliability and utility of ANAM UltraMobile without effective mitigation strategies. ANAM’s developers attempt to mitigate this by certifying few tablet models (see Table 1) with similar RT errors. However, as our study showed, a change in the OS from Android 11 to Android 12 on the same tablet model had a large and clinically meaningful effect on RT error. This would require limiting administration to tablet models with similar RT errors and taking steps to prevent software and OS updates. Limiting test administration to specific devices might be a barrier to large-scale military use due to potential supply challenges when attempting to purchase large quantities of obsolete equipment. Utilizing obsolete OSs can also increase security vulnerabilities, limit integration into electronic record systems, and impede implementation throughout the DoD. Conversely, while it is prudent to maintain current security standards through OS updates, keeping pace with Android OS updates will likely require time-intensive and routine RT error testing on various devices capable of administering ANAM UltraMobile.

The intra-device variability found in this study is consistent with prior research on laptop-based ANAM RT measurement accuracy (Arrieux et al., 2020), which showed variability between the Dell D630 and other comparable laptops. To manage such variability, ANAM’s developers created ANAM Data Converter software, which identifies the laptop specifications (e.g., model, number of cores, OS) and then automatically adjusts RTs to align with those recorded on the Dell D630 (Vista Life Sciences, 2015). Currently, a similar Data Converter for tablet specifications is not available for ANAM UltraMobile. Given the observed variability in RT errors across different tablet models and OSs, developing similar mitigation strategies is a crucial step to ensure the test’s reliability and clinical value. Ongoing research is essential for adapting to new sources of variability introduced by device updates.

### Limitations

This study used a robotic device to respond to stimuli and did not involve interaction with human participants. As described in the Methods section, while using the BBTK RKA offers precision at the microsecond level, software glitches and wearing of the RKA’s foam pad resulted in a small number of unusable trials (10% of SRT and 9% of SR2). Subtests were repeated when a subtest was missing responses to achieve the target number of trials (i.e., four administrations per interval for each subtest). Therefore, minor variability from the BBTK warrants some caution in interpreting these findings and offers the potential for replication studies using other RT measurement platforms.

Furthermore, while the study was designed based on prior research involving RT measurement accuracy of the laptop version of ANAM that used four administrations per subtest per device (Arrieux et al., 2020) and RT accuracy measurement on touchscreen tablet devices that used four preset RT intervals (Schatz et al., 2015), the selection of the number of subtests administered was somewhat arbitrary. However, without a clear precedent, the study sought to extend upon prior work on ANAM laptop RT accuracy (Arrieux et al., 2020) by including four different preset RT intervals for each subtest and including a larger number of trials at each RT interval reported by Schatz et al. (2015).

This study is also limited due to the proprietary nature of the ANAM UltraMobile software, which limits the disclosure of non-published technical reports or presentations by the test developer. Additionally, regarding the potential psychometric implications of the residual RT following adjustments, the differentials imply that examinees perform faster or slower on some ANAM UltraMobile subtests. However, the developer’s proposed adjustments do not account for accuracy scores used to calculate the throughput metric, the primary score for interpretive purposes. Any attempts to evaluate the psychometric equivalence of ANAM4-TBI-MIL and ANAM UltraMobile are incomplete without human performance data, ideally utilizing standardized throughput scores to interpret the results of a repeated measures counterbalanced study design in healthy and injured SMs. Finally, measurement error found in this study is not unique to ANAM UltraMobile, as measurement error is common across all cognitive tests. While the limitations are notable and results should be critically reviewed, the results of this study provide a valuable first step into determining the potential differences between ANAM UltraMobile and ANAM4-TBI-MIL and the clinical relevance of such differences.

## CONCLUSION

This study found that ANAM UltraMobile, which operates on Android tablets, recorded slower RTs than the laptop version. Additionally, RT errors varied considerably between tablet models and were psychometrically significant, potentially leading to incorrect clinical interpretations. The study also found previously undisclosed prototype RT equating adjustments automatically applied by ANAM UltraMobile software, appearing as variables in the summary data files generated for each administration of each ANAM subtest. These analyses revealed that RT adjustments were universally applied, regardless of device specifications. These inconsistencies highlight the need for further research to examine the psychometric properties of equated scores.

While the findings from this study indicate a potential for interpretive error, RT measurement accuracy might be negligible when interpreting performance on subtests with longer average RTs or assessing overall performance across the ANAM battery. Further, the magnitude of clinically meaningful differences between performance on laptop and tablet platforms will be examined in future analyses. The full psychometric implications of the equating adjustments cannot be evaluated or appraised until human performance data are available in the next phase of the study, which is currently in progress. Until then, adjusted ANAM UltraMobile scores should not be compared with the existing laptop-derived military normative data or other laptop-based interpretive criteria, such as reliable change parameters or performance validity indicators.

## Supplementary Material

ANAM_UltraMobile_RTR1_supplemental_table_1_20240606_arclin_acae070

Supplemental_Figure_1_300_dpi_tiff_arclin_acae070

Supplemental_Figure_1_caption_arclin_acae070

## Disclaimer

The views expressed in this manuscript are those of the authors and do not necessarily represent the official policy or position of the Defense Health Agency, Department of Defense, or any other U.S. government agency. This work was prepared under Contract HT0014-22-C-0016 with DHA Contracting Office (CO-NCR) HT0014 and, therefore, is defined as U.S. Government work under Title 17 U.S.C.§101. Per Title 17 U.S.C.§105, copyright protection is not available for any work of the U.S. Government. For more information, please contact dha.TBICOEinfo@health.mil.
