# Supplementary material for: Accuracy of Reaction Time Measurement on Automated Neuropsychological Assessment Metric UltraMobile
Source: Arch Clin Neuropsychol. 2024 Sep 13;40(2):310–8. doi: 10.1093/arclin/acae070 (PMC11836681; doi:10.1093/arclin/acae070)
Supplement: ANAM_UltraMobile_RTR1_supplemental_table_1_20240606_arclin_acae070 [file anam_ultramobile_rtr1_supplemental_table_1_20240606_arclin_acae070.docx]

Supplemental Table 1. Psychometric implications of observed tablet RT error and proposed ANAM UltraMobile equating adjustments

| ANAM subtest | ANAM UltraMobile RT equating adjustments  (ms) | Implied ANAM UltraMobile RT performance differential  (ms) ^a^ | SDs for RTC from 2019 laptop norms | Implied ANAM UltraMobile performance differentials  (z-score) ^b^ | Range of total laptop-based ANAM4 RT performance differentials  (ms) ^c^ | Range of total laptop-based ANAM4 RT performance differentials  (z-score) ^d^ | Range of implied ANAM UltraMobile RT performance differentials  (ms) ^e^ | Range of ANAM UltraMobile RT performance differentials not accounted  (z-score) ^f^ |
| --- | --- | --- | --- | --- | --- | --- | --- | --- |
| SRT | -20 | +20 | 46.04 | +0.43 | -89.61 to -19.25 | -1.95 to -0.42 | -69.61 to +0.75 | -1.51 to +0.02 |
| SR2 | -40 | +40 | 44.12 | +0.91 | -89.61 to -19.25 | -2.03 to -0.44 | -49.61 to +20.75 | -1.12 to +0.47 |
| ST6 | +66 | -66 | 198.25 | -0.33 | -155.61 to -85.25 | -0.78 to -0.43 | -89.61 to -19.25 | -0.45 to -0.10 |
| SPD | +40 | -40 | 525.79 | -0.08 | -129.61 to -59.25 | -0.25 to -0.11 | -89.61 to -19.25 | -0.17 to -0.04 |
| PRO | +30 | -30 | 89.46 | -0.34 | -119.61 to -49.25 | -1.34 to -0.55 | -89.61 to -19.25 | -1.00 to -0.22 |
| MTH | +45 | -45 | 811.87 | -0.06 | -134.61 to -64.25 | -0.17 to -0.08 | -89.61 to -19.25 | -0.11 to -0.02 |
| M2S | +125 | -125 | 474.96 | -0.26 | -214.61 to -144.25 | -0.45 to -0.3 | -89.61 to -19.25 | -0.19 to -0.04 |
| GNG | -53 | +53 | 30.05 | +1.76 | -89.61 to -19.25 | -2.98 to -0.64 | -36.61 to +33.75 | -1.22 to +1.12 |
| CDS | +120 | -120 | 274.38 | -0.44 | -209.61 to -139.25 | -0.76 to -0.51 | -89.61 to -19.25 | -0.33 to -0.07 |
| CDD | +100 | -100 | 367.09 | -0.27 | -189.61 to -119.25 | -0.52 to -0.32 | -89.61 to -19.25 | -0.24 to -0.05 |

Range of observed mean RT error from both SRT tests across all tablets relative to laptops (ms) +19.25 to +89.61. ^a^ Relative to laptop-based ANAM,  ^b^ As a proportion of laptop normative RTC SDs. ^c^ Relative to ANAM UltraMobile on various tablets based on +/- equating adjustments combined with reversed RT error. ^d^ Relative to ANAM UltraMobile as a proportion of laptop-based normative RTC SDs. ^e^ Relative to laptop-based ANAM4 unaccounted for by current equating adjustments. ^f^ As a proportion of laptop-based normative RTC SD
