# Supplementary figures and images for: Accuracy of Reaction Time Measurement on Automated Neuropsychological Assessment Metric UltraMobile
Source: Arch Clin Neuropsychol. 2024 Sep 13;40(2):310–8. doi: 10.1093/arclin/acae070 (PMC11836681; doi:10.1093/arclin/acae070)

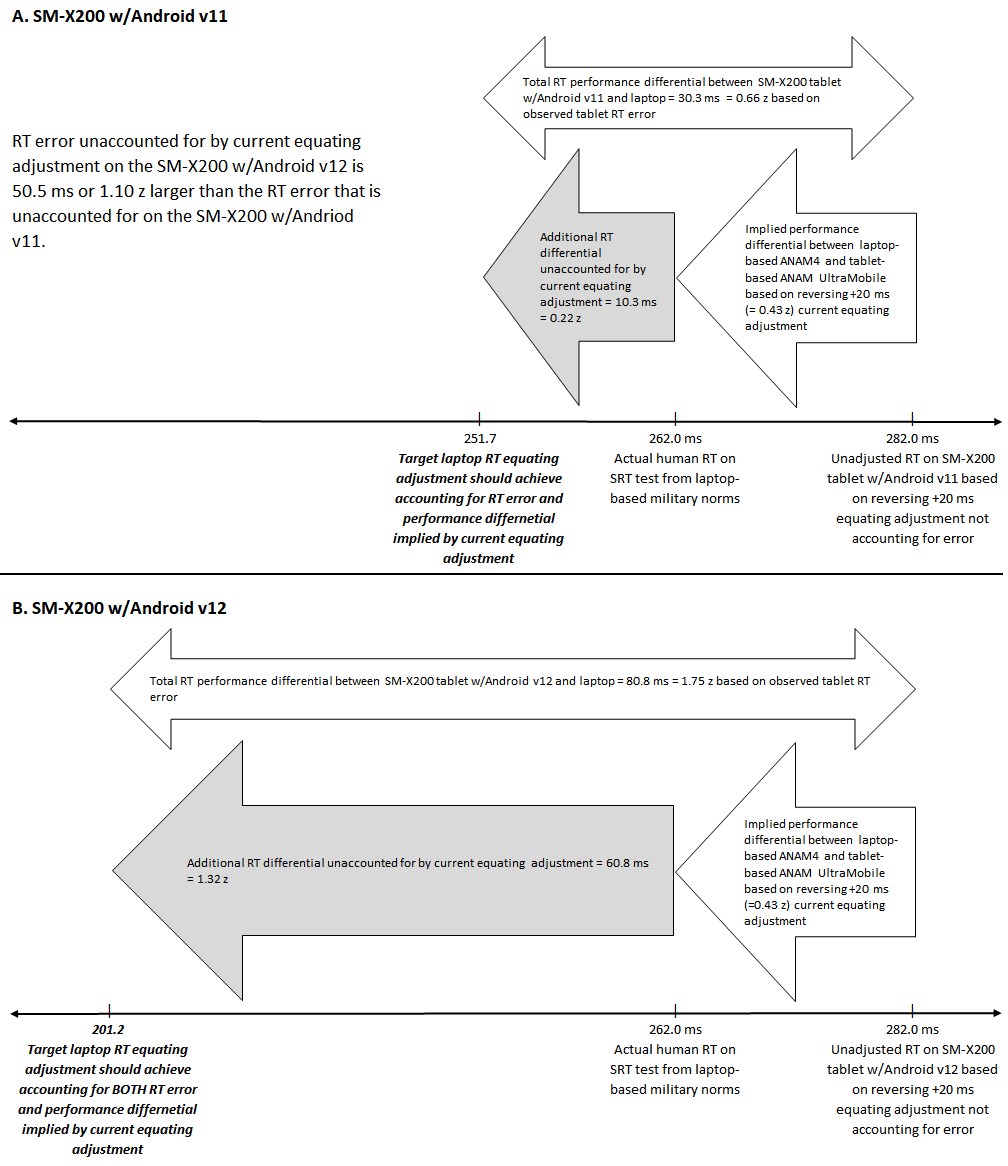

Supplement: Supplemental_Figure_1_300_dpi_tiff_arclin_acae070 [file supplemental_figure_1_300_dpi_tiff_arclin_acae070.jpeg]
