# Supplementary material for: Accuracy of Reaction Time Measurement on Automated Neuropsychological Assessment Metric UltraMobile
Source: Arch Clin Neuropsychol. 2024 Sep 13;40(2):310–8. doi: 10.1093/arclin/acae070 (PMC11836681; doi:10.1093/arclin/acae070)
Supplement: Supplemental_Figure_1_caption_arclin_acae070 [file supplemental_figure_1_caption_arclin_acae070.docx]

Supplemental Figure 1. Comparison of RT performance differentials and equating adjustments for ANAM UltraMobile’s SRT test between SM-X200 tablets running Android v11 and v12
